# Supplementary material for: Effects of Chronic High-Frequency rTMS Protocol on Respiratory Neuroplasticity Following C2 Spinal Cord Hemisection in Rats
Source: Biology (Basel). 2022 Mar 19;11(3):473. doi: 10.3390/biology11030473 (PMC8945729; doi:10.3390/biology11030473)
Supplement: Supplementary file 1 [file biology-11-00473-s001.zip › biology-1618001-supplementary.pdf]

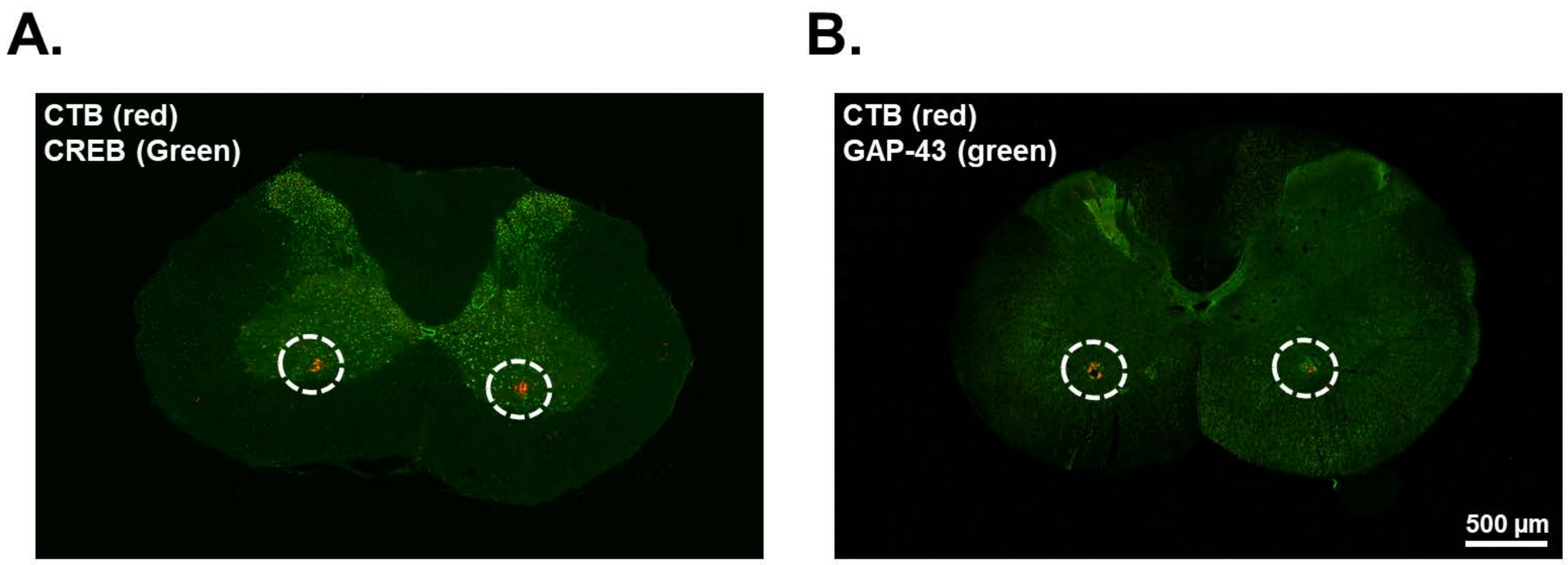

Figure S1: Representative C3-C6 spinal cord sections labeled for CTB and CREB or GAP-43
A. Representative image showing the phrenic motoneurons labeling in red and CREB labeling in green in the C3-C6 spinal cord. **B.** Representative image showing the phrenic motoneurons labeling in red and GAP-43 labeling in green in the C3-C6 spinal cord.

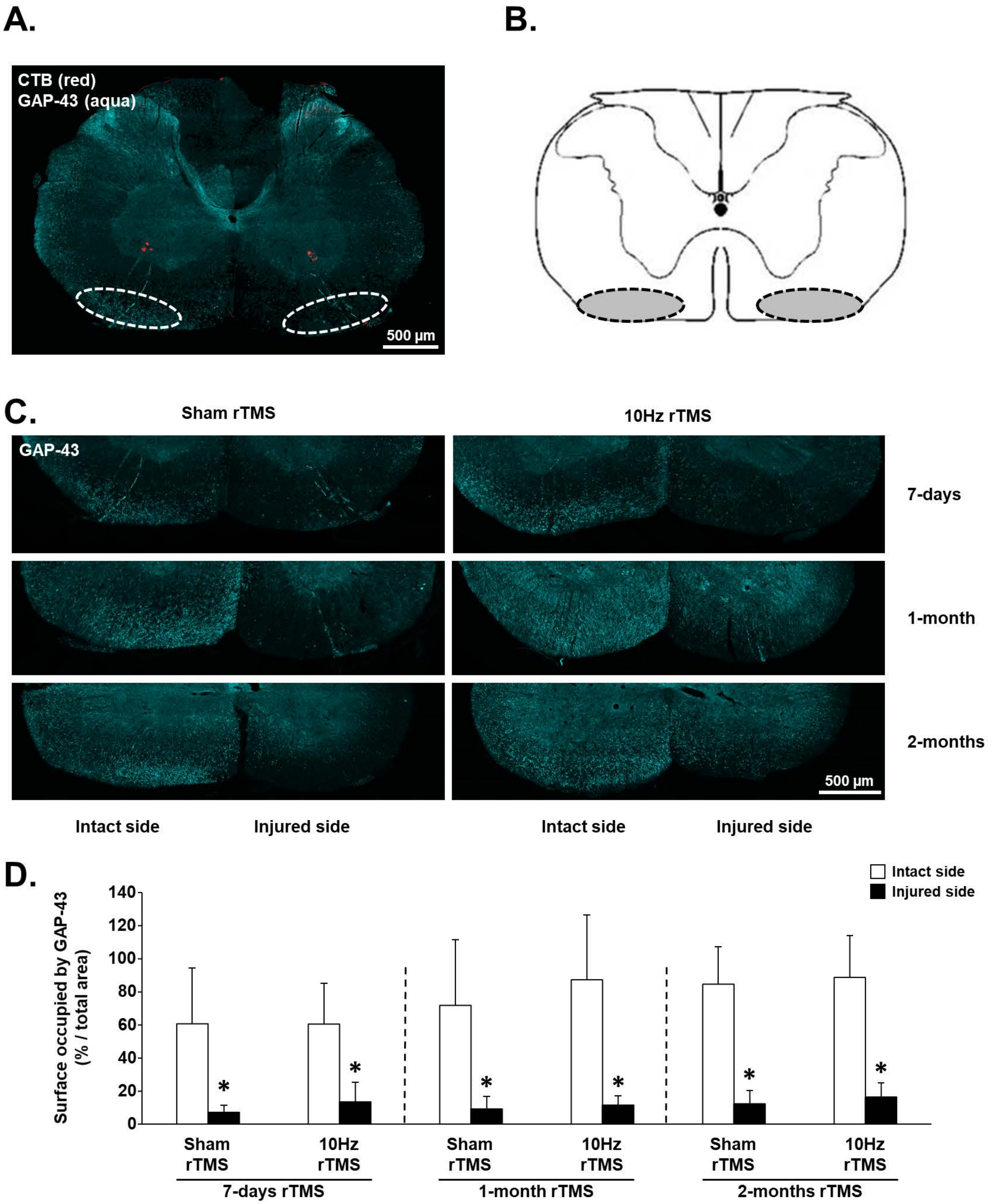

Figure S2: GAP-43 expression in C3-C6 ventrolateral funiculi following C2 hemisection

A. Representative image showing location of phrenic motoneurons in red and the location of the ventrolateral funiculi (white dashed line). **B**. Schematic representation of a transversal section of the spinal cord showing the ventrolateral funiculi (black dashed line). **C.** Representative images showing expression of GAP-43 expression in C3-C6 ventrolateral funiculi in C2 hemisected rats, following 7-days, 1-month or 2-months Sham or 10Hz rTMS treatment. **D.** Quantitative analysis of the GAP-43 immunolabeled area in the ventrolateral funiculi for intact and injured sides of Sham or 10Hz rTMS treated C2 hemisected animals following 7-days, 1-month or 2 months treatment. There is no difference between intact side groups, and between injured groups (T-test, $p>0.05$). * compared to intact side, $p<0.01$.

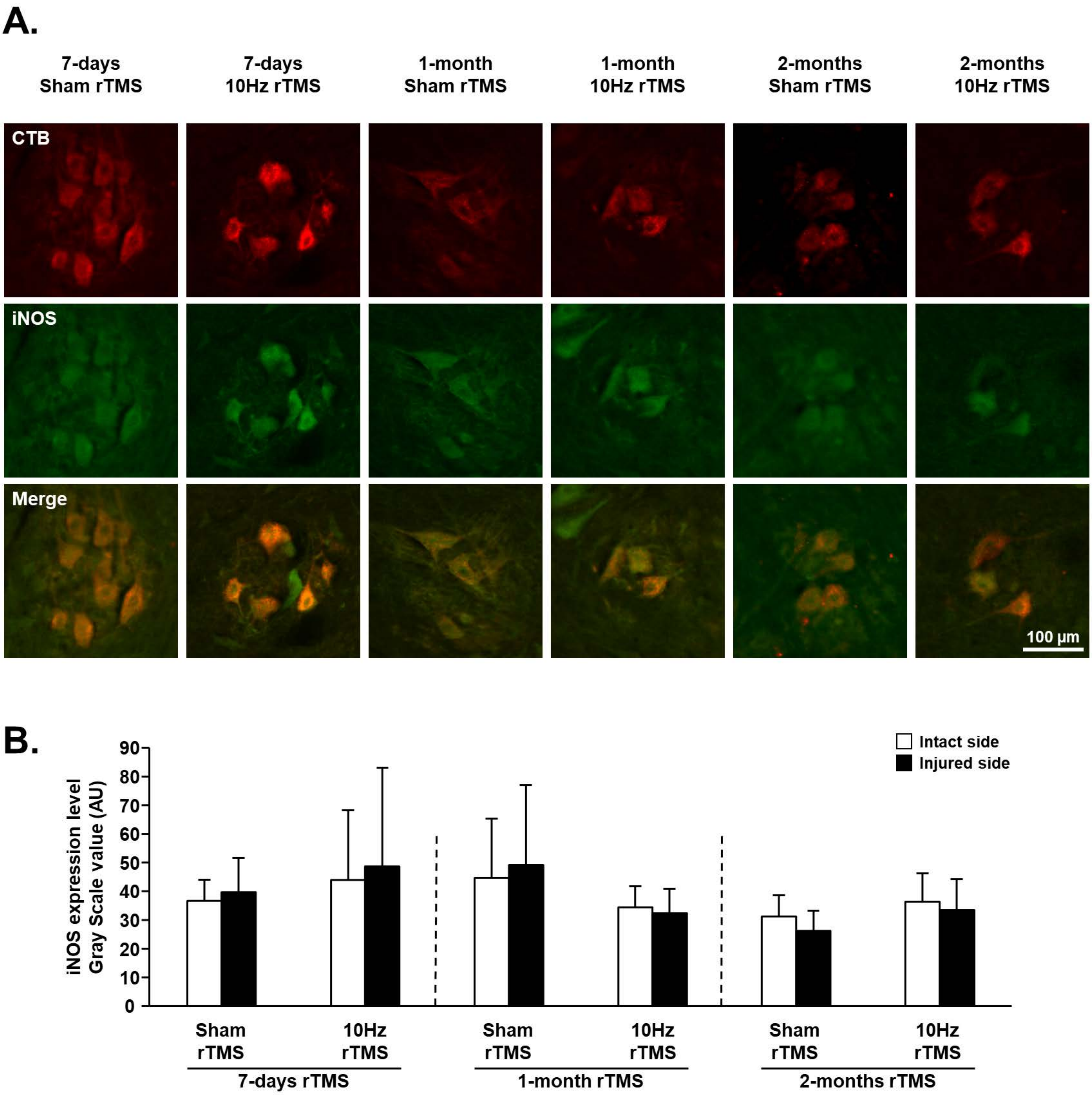

Figure S3: **iNOS expression in phrenic motoneurons following C2 hemisection**

A. Representative images showing expression of iNOS in intact side phrenic motoneurons labeled with CTB in C2 hemisected rats, following 7-days, 1-month or 2-months Sham or 10Hz rTMS treatment. **B.** Quantification of iNOS expression in phrenic motoneurons for intact and injured sides of Sham or 10Hz rTMS treated C2 hemisected animals following 7-days, 1-month or 2 months treatment. There is no difference between the intact and the injured sides for the different groups (Paired t-test for intact *vs* injured side, p>0.05 There is no difference between intact side groups, and between injured groups (T-test, p>0.05).

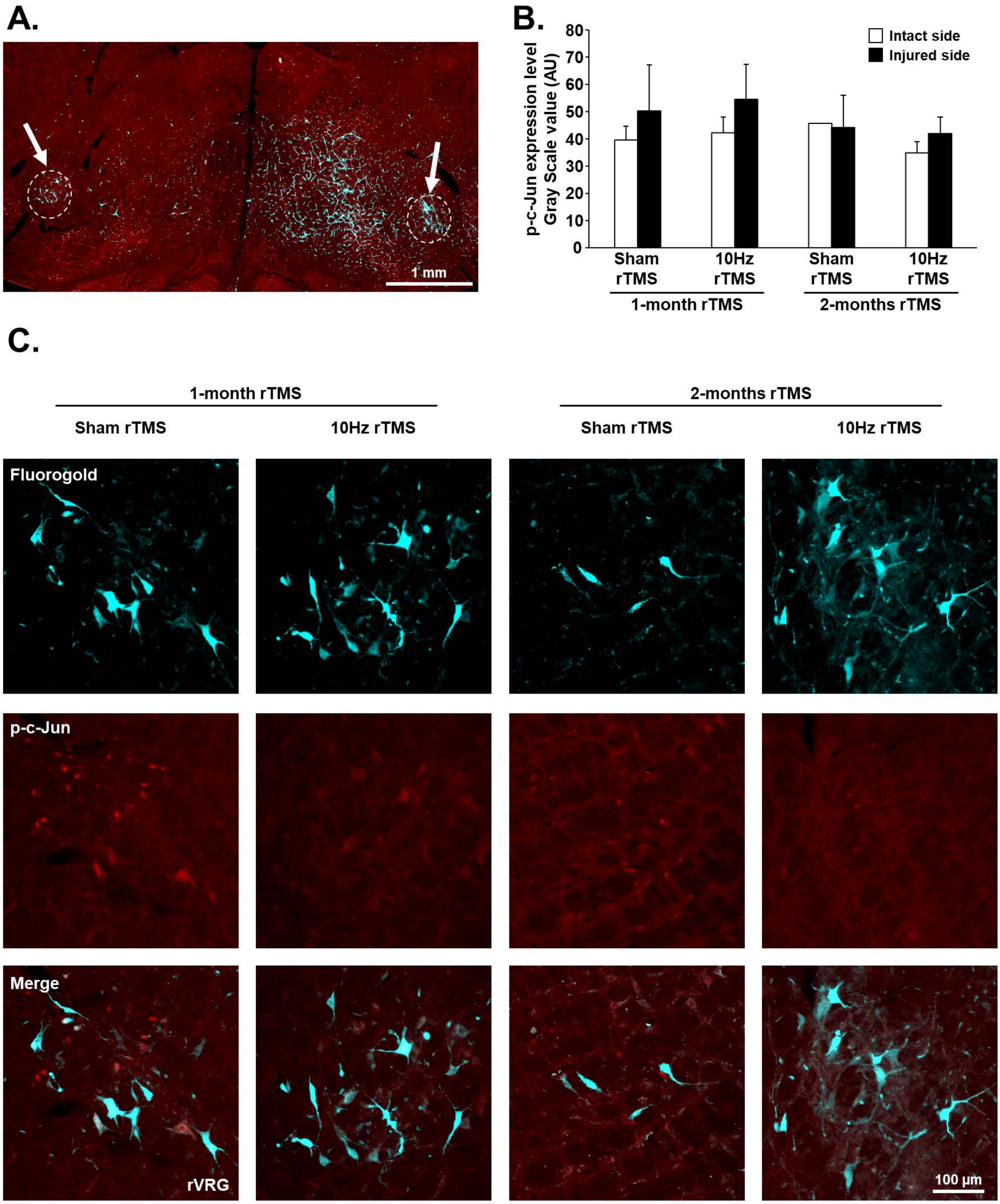

Figure S4: **p-c-Jun expression in injured rVRG neurons**.
A. Representative images showing rVRG in the brainstem. rVRG neurons are labeled with Fluorogold. **B.** Quantification of p-c-Jun expression in injured rVRG neurons following 1-month and 2-months 10Hz rTMS or Sham rTMS. **C**. representative images of p-c-Jun expression in inured rVRG neurons following 1-month and 2-months 10Hz rTMS or Sham rTMS. There is no difference between the different groups (t-test when possible, $p>0.05$).

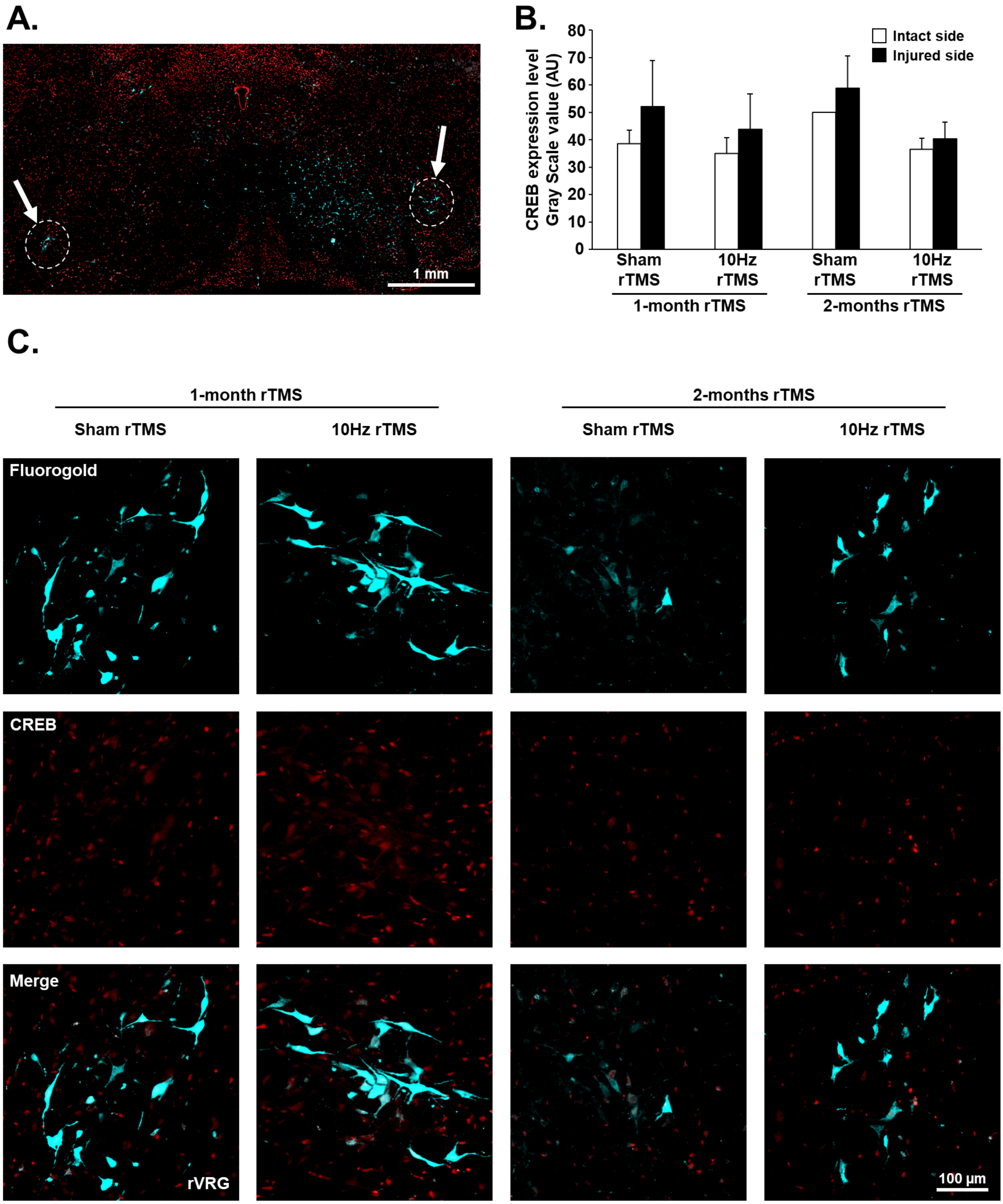

Figure S5: **CREB expression in injured rVRG neurons**.

A. Representative images showing rVRG in the brainstem. rVRG neurons are labeled with Fluorogold. **B.** Quantification of CREB expression in injured rVRG neurons following 1-month and 2-months 10Hz rTMS or Sham rTMS. **C.** representative images of CREB expression in inured rVRG neurons following 1-month and 2-months 10Hz rTMS or Sham rTMS. There is no difference between the different groups (t-test when possible, p>0.05).

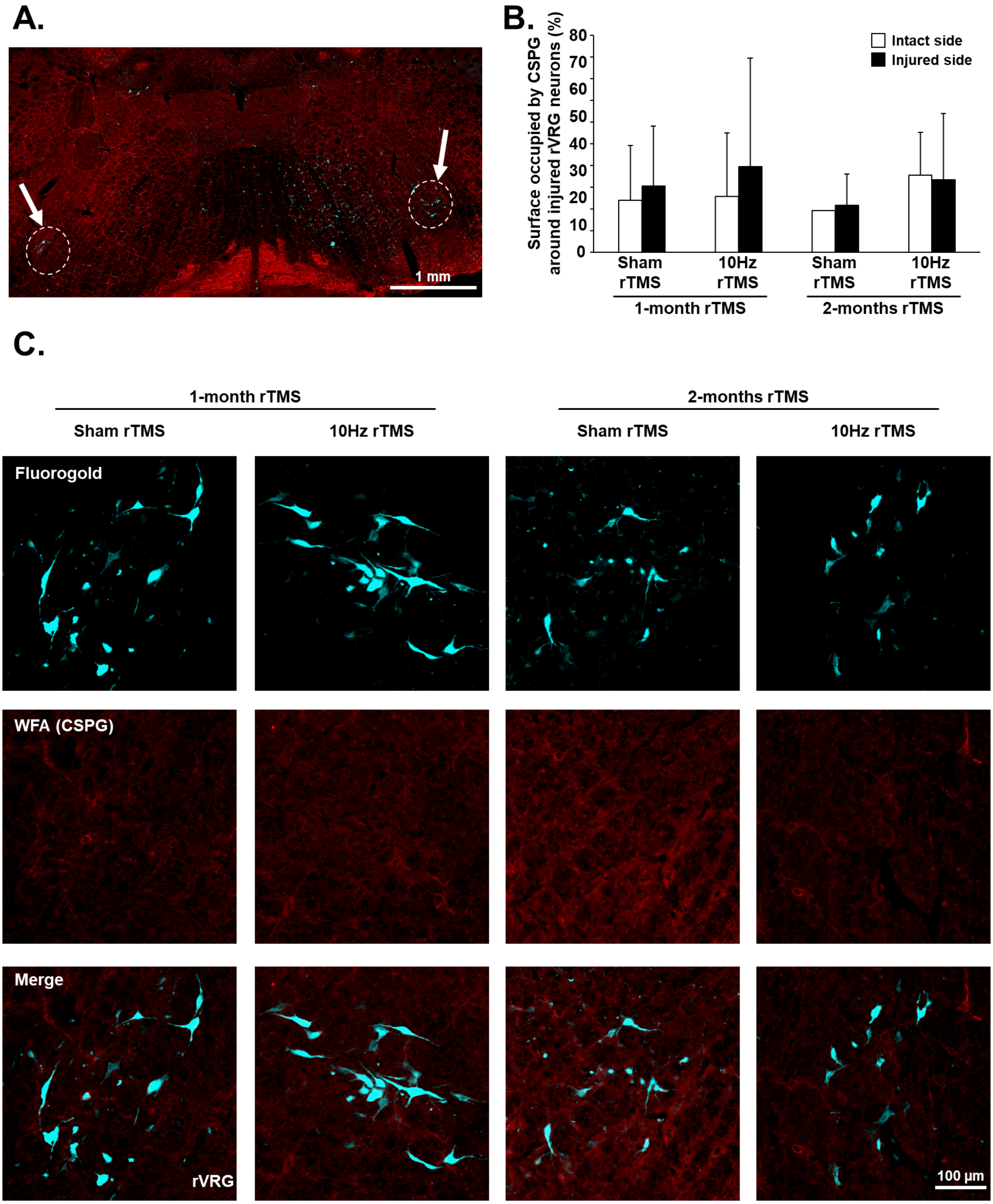

Figure S6: CSPG expression in in the area around injured rVRG neurons.
A. Representative images showing rVRG in the brainstem. rVRG neurons are labeled with Fluorogold. **B.** Quantification of CSPG expression in the area around injured rVRG neurons following 1-month and 2-months 10Hz rTMS or Sham rTMS. **C**. representative images of CSPG expression in the area around inured rVRG neurons following 1-month and 2-months 10Hz rTMS or Sham rTMS. There is no difference between the different groups (t-test when possible, p>0.05).

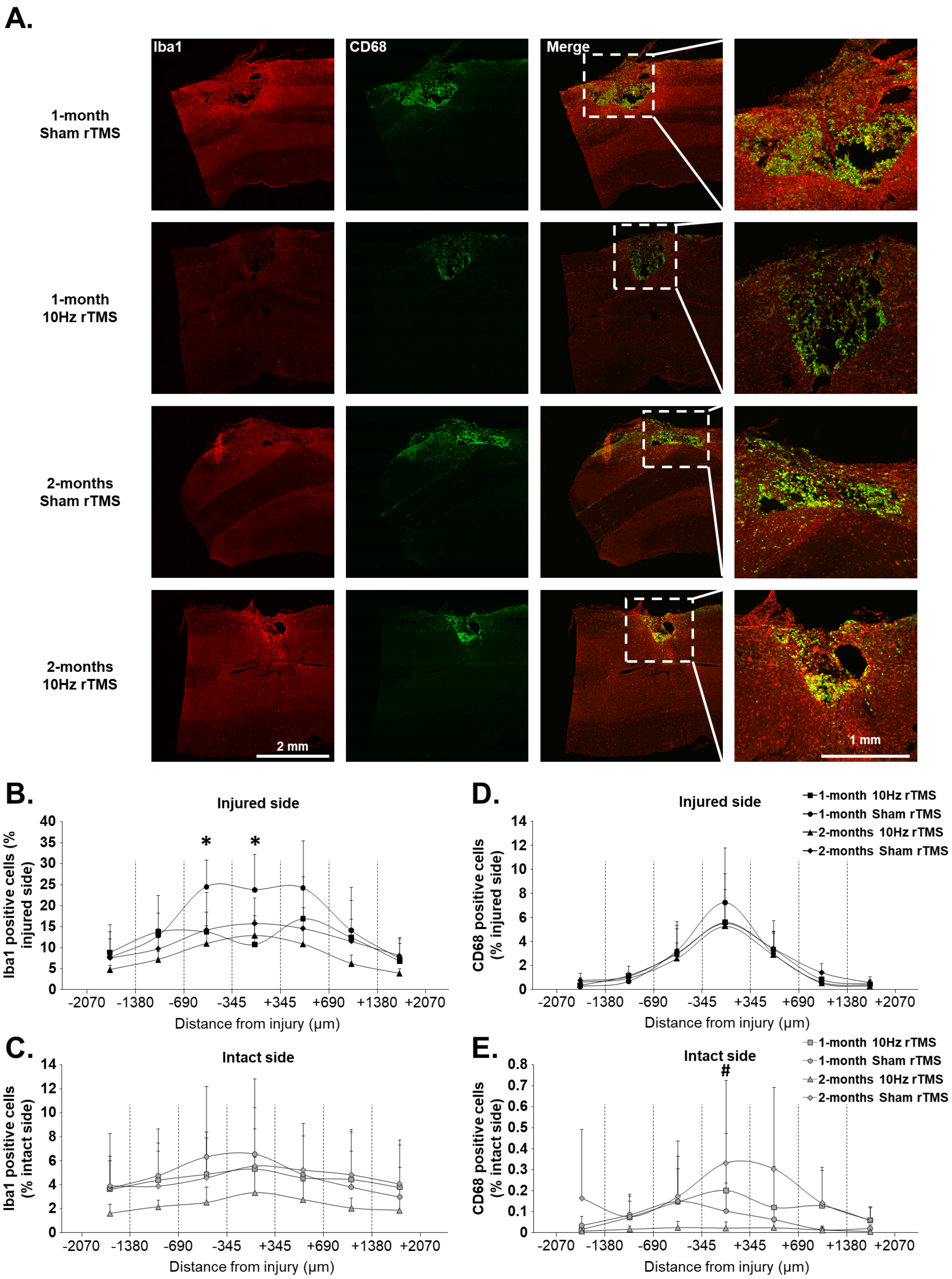

Figure S7 : Iba1 and CD68 expression in the C1-C3 spinal cord following C2 hemisection

A. Representative images showing expression of Iba1 and CD68 at the site of the lesion in C2 hemisected rats, following 1-month or 2-months Sham or 10Hz rTMS treatment. Quantification of Iba1 expression intact side (**B**) or injured side (**C**) of Sham or 10Hz rTMS treated C2 hemisected animals following 1-month or 2 months treatment. Quantification of CD68 expression intact side (**D**) or injured side (**E**) of Sham or 10Hz rTMS treated C2 hemisected animals following 1-month or 2-months treatment. * 1-month 10 Hz rTMS *vs* 1-month Sham Hz rTMS, Two-way-ANOVA, $p<0.05$. # 2-months 10 Hz rTMS *vs* 2-months Sham Hz rTMS, Two-way-ANOVA, $p<0.05$.

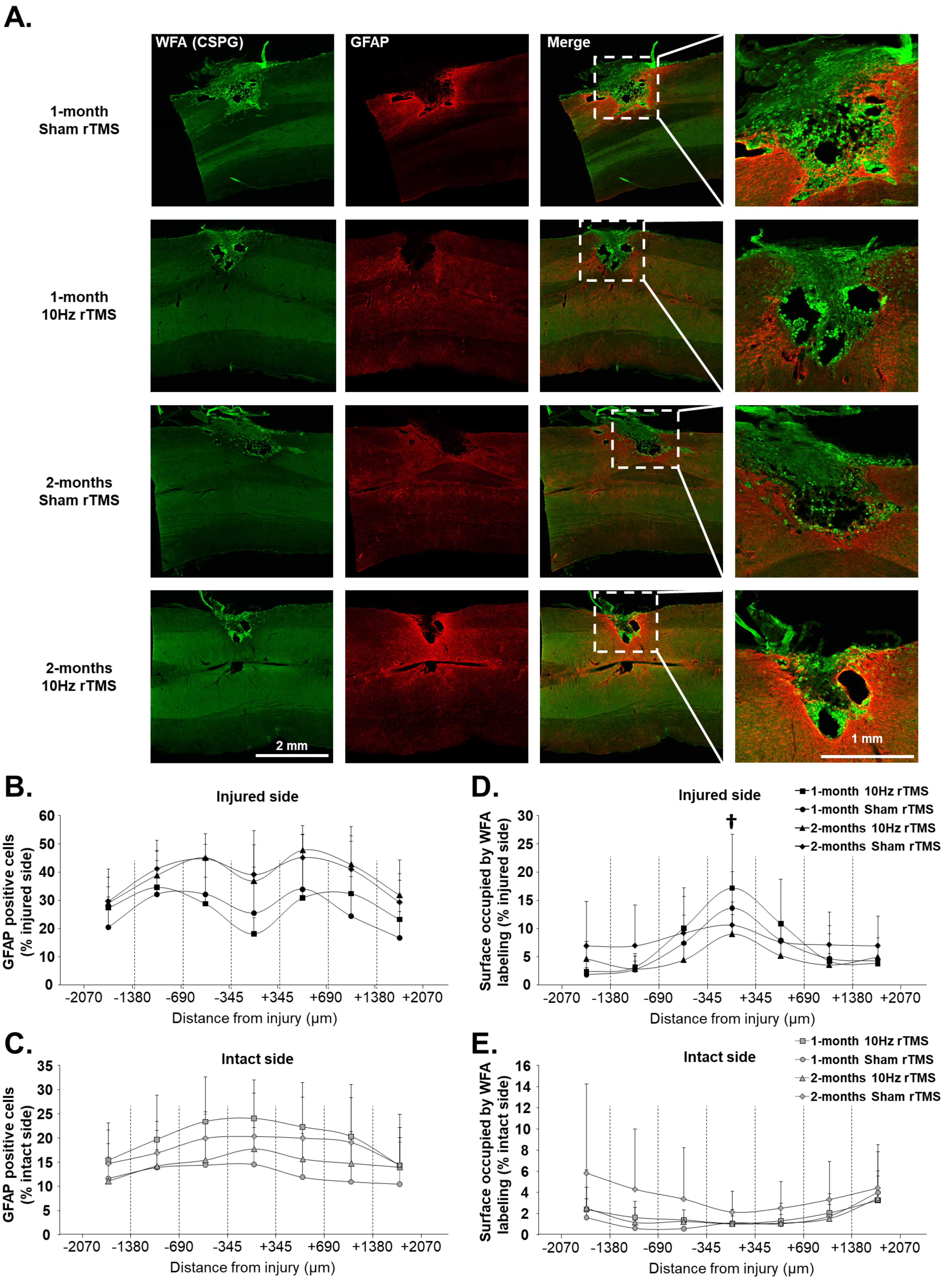

Figure S8 : GFAP and WFA expression in the C1-C3 spinal cord following C2 hemisection

A. Representative images showing expression of GFAP and WFA (CSPG) at the site of the lesion in C2 hemisected rats, following 1-month or 2-months Sham or 10Hz rTMS treatment. Quantification of Iba1 expression intact side (**B**) or injured side (**C**) of Sham or 10Hz rTMS treated C2 hemisected animals following 1-month or 2-months treatment. Quantification of CD68 expression intact side (**D**) or injured side (**E**) of Sham or 10Hz rTMS treated C2 hemisected animals following 1-month or 2-months treatment. † 1-month 10 Hz rTMS *vs* 2-months 10 Hz rTMS, Two-way-ANOVA, $p<0.05$.
